# Laser Powder Bed Fusion of Ti-6Al-2Sn-4Zr-6Mo Alloy and Properties Prediction Using Deep Learning Approaches

**DOI:** 10.3390/ma14082056

**Published:** 2021-04-19

**Authors:** Hany Hassanin, Yahya Zweiri, Laurane Finet, Khamis Essa, Chunlei Qiu, Moataz Attallah

**Affiliations:** 1 School of Engineering, Technology and Design, Canterbury Christ Church University, Canterbury CT1 1QU, UK; 2 Faculty of Science, Engineering and Computing, Kingston University London, London SW15 3DW, UK; y.zweiri@kingston.ac.uk; 3 Khalifa University Center for Autonomous Robotic Systems, Department of Aerospace Engineering, Khalifa University of Science and Technology, Abu Dhabi P.O. Box 127788, United Arab Emirates; 4 School of Materials and Metallurgy, University of Birmingham, Birmingham B15 2TT, UK; l.finet@bham.ac.uk (L.F.); k.e.a.essa@bham.ac.uk (K.E.); m.m.attallah@bham.ac.uk (M.A.); 5 Department of Materials Processing Engineering and Automation, Beihang University, Xueyuan Road, Haidian District, Beijing 100191, China; chunlei_qiu@buaa.edu.cn

**Keywords:** deep learning, additive manufacturing, porosity, powder bed fusion

## Abstract

Ti-6Al-2Sn-4Zr-6Mo is one of the most important titanium alloys characterised by its high strength, fatigue, and toughness properties, making it a popular material for aerospace and biomedical applications. However, no studies have been reported on processing this alloy using laser powder bed fusion. In this paper, a deep learning neural network (DLNN) was introduced to rationalise and predict the densification and hardness due to Laser Powder Bed Fusion of Ti-6Al-2Sn-4Zr-6Mo alloy. The process optimisation results showed that near-full densification is achieved in Ti-6Al-2Sn-4Zr-6Mo alloy samples fabricated using an energy density of 77–113 J/mm^3^. Furthermore, the hardness of the builds was found to increase with increasing the laser energy density. Porosity and the hardness measurements were found to be sensitive to the island size, especially at high energy density. Hot isostatic pressing (HIP) was able to eliminate the porosity, increase the hardness, and achieve the desirable α and β phases. The developed model was validated and used to produce process maps. The trained deep learning neural network model showed the highest accuracy with a mean percentage error of 3% and 0.2% for the porosity and hardness. The results showed that deep learning neural networks could be an efficient tool for predicting materials properties using small data.

## 1. Introduction

Additive manufacturing (AM) and deep learning (DL) are two critical pillars of Industry 4.0, which transforms the manufacturing industry’s paradigm. Additive manufacturing allows mechanical parts production with a high degree of complexity based on the incremental layer-by-layer concept [1,2,3,4]. Laser powder bed fusion (LPBF) has been widely considered in a wide range of industries such as biomedical [5,6,7,8], aerospace [9,10,11,12], and automotive [13], as it is able to build components with high quality from different materials such as metals, ceramics, and polymers [14,15,16,17,18]. In this technique, a fast-moving laser beam is employed as an energy source to scan and selectively melt the metal powder, resulting in the production of dense metal parts. LPBF technology can significantly change the manufacturing of metal alloys making it more efficient, cost-effective, and material saving. Typically, the influence and optimisation of AM processes’ process parameters are carried out using statistical approaches such as the design of experiments (DOE). Although these techniques proved to be efficient, a typical drawback is that the AM process parameters were assumed as static, without considering that AM is regarded as a dynamic process [19,20]. Particularly, AM has a temporal nature characterised by scattered results due to repeated heating and cooling cycles, inter-layer interactions, variation in the heat distribution within the building platform, and the oxygen level fluctuation even if it is within a specific range [21]. Finite element modelling and computational fluid dynamics are two numerical techniques that have been successfully implemented to predict manufactured parts’ behaviour. Belhocine et al. successfully developed a numerical simulation of structural analysis and a transient thermal coupled thermo-structural method of brake disc rotors manufactured using different materials [22]. The unavailability of research studies considering the above-mentioned issues motivates the research direction to other techniques. Deep learning (DL) has been advanced in the past few years and has enabled new opportunities for the analytical studies of AM [23]. However, DL is efficient with big data, and the small data nature of AM experiments is a barrier towards the use of this technology in the analytical modelling of AM processes [24].

Artificial neural networks are mathematical algorithms inspired by the neural networks of animal brains. A shallow neural network (SNN) consists of one input layer, one hidden layer, and one output layer. A neural network with more than two layers is referred to as deep learning neural networks (DLNNs) [25]. DLNNs are more efficient than SNNs when modelling complex problems as it uses nonlinear activation functions at several layers [26]. The greedy layer-wise pre-training technique proves to be effective in overcoming local minima [27,28]. Greedy layer-wise pre-training sets the weights of a neural network to values in the neighbourhood of a local minimum. Hence, it aids the optimisation process and induces better model generalisation. A stacked auto-encoder (SAE) architecture was used as an alternative to the Boltzmann machine in a pre-training approach [29].

Titanium alloys are essential materials for the aerospace and biomedical sectors because of their lightweight and superior mechanical properties. The two α+β titanium-based alloys are characterised by their ability to change the amount and distribution of untransformed phases by thermal treatments or ageing, which makes them account for approximately 25–30% of the weight of modern aeroplanes engines. Simultaneously, the figure goes up to about 50% for military jet engines [30]. Ti-6Al-2Sn-4Zr-6Mo is an α+β alloy with α-phase of a hexagonal closed packed crystal structure, in addition to its body-centred cubic β-phase. Ti-6Al-2Sn-4Zr-6Mo is characterised by its high fatigue strength and toughness properties at intermediate temperature level in the range of 315–400 °C, which makes it ideal for compressor disks, turbine blisks, spacers, and seals [31,32]. Ti-6Al-4V is one of the most researched titanium alloys using AM, which can be considered a benchmark because it is used in many applications, including aerospace and biomedical industries. Many of the efforts on using AM to process Ti-6Al-4V have been dedicated to processing microstructure relationships to control the mechanical properties and to minimise the inherited defects of LPBF [33,34,35]. Owing to the layer-by-layer concept of LPBF, the poor surface roughness of Ti-6Al-4V alloy is among the issues that have been investigated [36]. Other studies include the use of post-heat treatments such as annealing [37], stress-relieving [38], solution treatment [39], and hot isostatic pressing (HIP) [40] to improve the microstructure of the as LPBF samples and to reduce the developed defects. Studies on using LPBF to process other titanium alloys such as Ti-24Nb-4Zr-8Sn [41], Ti-13Nb-13Zr [42], and Ti-6Al-7Nb [39] have also been carried out by several research groups. Literature review showed that other alloys such as Ti-6Al-2Sn-4Zr-6Mo had not benefited from the comprehensive investigation using LPBF compared to other alloys such as Ti-6Al-4V alloy. Therefore, this paper’s novelty is to introduce the processing and the characterisation of Ti-6Al-2Sn-4Zr-6Mo alloy using LPBF. The study addresses the influence of the LPBF process parameters on the properties of the Ti-6Al-2Sn-4Zr-6Mo alloy. In addition, the study also developed several deep learning models for process optimisation of the measured data of that alloy, aiming to predict the properties of the developed alloy when processed using LPBF. The process parameters under this study include laser power, scanning speed, island size, and hatching spacing. The porosity, hardness, and microstructure developments at various LPBF processing parameters were studied and modelled using deep learning. Shallow neural network supervised training, deep neural network supervised training, and deep learning neural network unsupervised greedy layer-wise pre-training approaches were implemented to understand the influence of the LPBF process parameters on the properties of the Ti-6Al-2Sn-4Zr-6Mo alloy and to enable the accurate prediction of the properties of that alloy when processed by LPBF.

## 2. Materials and Methods

**Figure 1:**
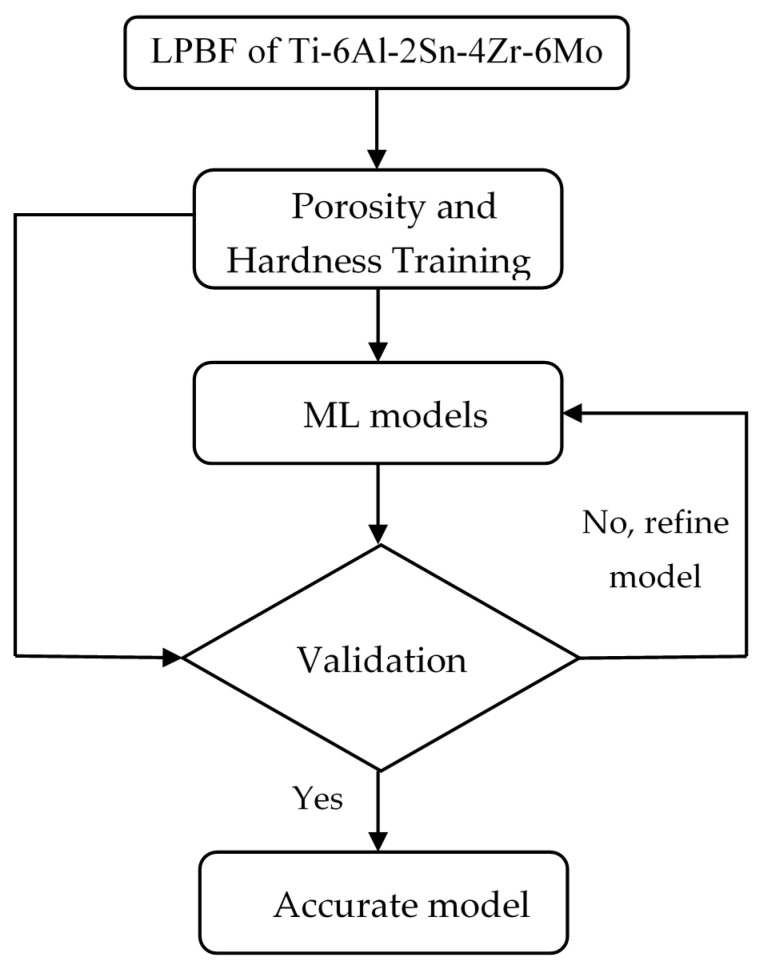
The flow diagram of the processing and modelling procedure.

The flow diagram of the method of processing Ti-6Al-2Sn-4Zr-6Mo and the development of different ML models is shown in Figure 1.

### 2.1. Samples Fabrication

**Figure 2:**
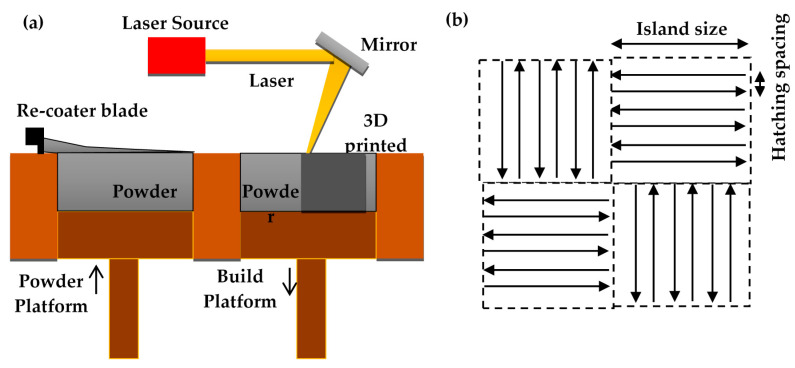
(**a**) A schematic diagram of the laser powder bed fusion system, (**b**) the island scanning and hatching spacing contours.

Ti-6Al-2Sn-4Zr-6Mo powder, supplied by (TLS, Bitterfeld, Germany) was sieved in the range of 20–50 µm. A laser powder bed fusion system (M2 Concept Laser, Lichtenfels Germany) equipped with Nd:YAG laser of a power up to 200 W and a laser speed up to 4000 mm/s was used to prepare 10 mm × 10 mm × 10 mm cuboid samples. A schematic diagram of the LPBF system is shown in Figure 2a. All the cuboid samples were made on a titanium building substrate in an Argon chamber with  O_2_ < 100 ppm. The island scanning method was used in which the laser-scanned section is divided into squares, known as islands, Figure 2b. In all experiments, 3 samples were built for each run to ensure repeatability.

Laser power in the range of (100–200 W), laser speed of (800–1800 mm/s), hatch spacing constant h_1_ of (0.2–0.8), and island size of (2–8 mm) were the input parameters used in the preparation of the matrix. The volumetric energy density (E) is one of the critical terms used in LPBF processes. It is an empirical parameter used to represent the effect of LPBF laser parameters on the samples’ properties. The equation of the volumetric energy density is shown in Equation (1) [43].

(1) $$E=\frac{P}{\nu \cdot h\cdot b}$$

where P is the power of the laser in watts, ν is the laser speed in mm/s, h hatching spacing in mm, and b is the powder layer thickness in mm. The generated matrix parameters and levels are listed in Table 1.

Hot isostatic pressing was carried in an EPSI HIP vessel at the University of Birmingham, which has a maximum temperature of 1450 °C and a maximum pressure of 200 × 10^6^ Pa. The HIP unit is a water-cooled vessel with molybdenum heating elements with a compressed Argon gas system. The HIP cycle used in this experiment is 800 °C/103 MPa/4 h followed by furnace cooling.

### 2.2. Microstructural and Mechanical Characterisation

The samples were cut vertically across the X-Z plane into two sections in order to obtain cross-sections of building layers. Metallographic samples were polished using the typical grinding and polishing methods. To characterise the samples’ porosity, the polished cross-sections were characterised using an optical microscope (OM) Zeiss Axioskop and (Peine, Germany) Hitachi TM300 (Hitachi, Japan) desktop electron scanning microscopy (SEM). More than 80 images were captured and stitched to construct most of the cross-section by using ImageJ (an image editor). The software was used to determine the fractional area of the porosity. Vickers micro-hardness characterisation was conducted using an INDENTEC hardness testing system (Brierley, UK) with an indenter load of 30 kg. X-ray diffraction (XRD) using Inel EQUINOX 3000 (Waltham, MA, USA), which has a Cu-fiber laser of 1.54 Å was also used to examine the phase evolution between the as SLMed and HIPed samples.

### 2.3. Deep Learning

**Figure 3:**
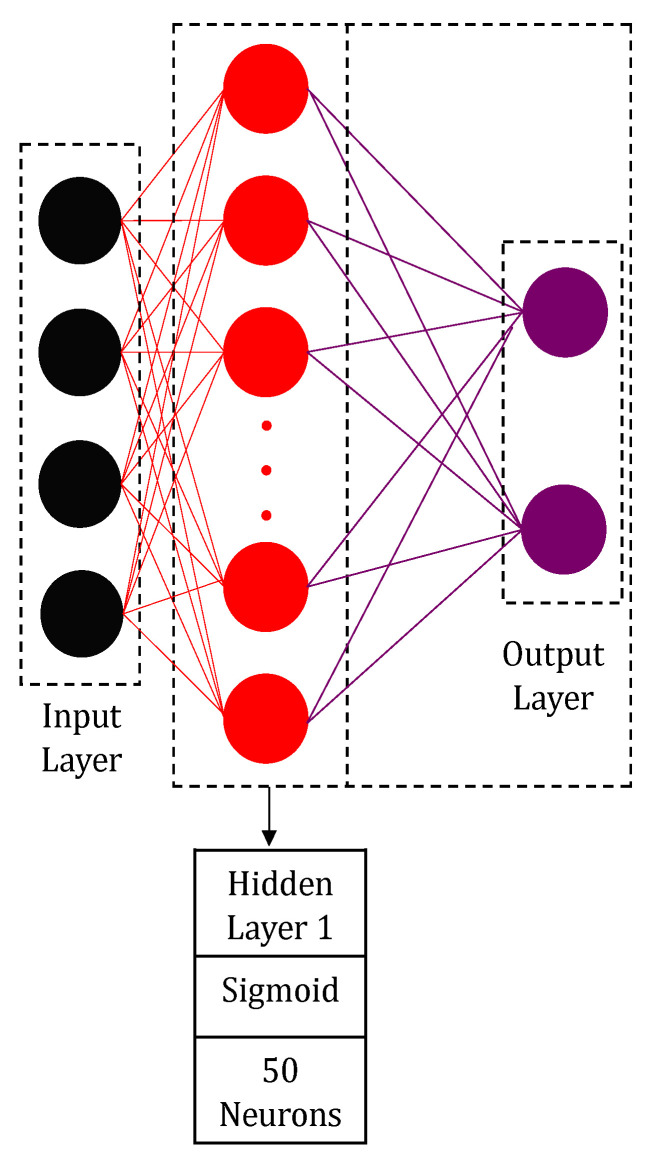
Shallow neural network supervised training.

**Figure 4:**
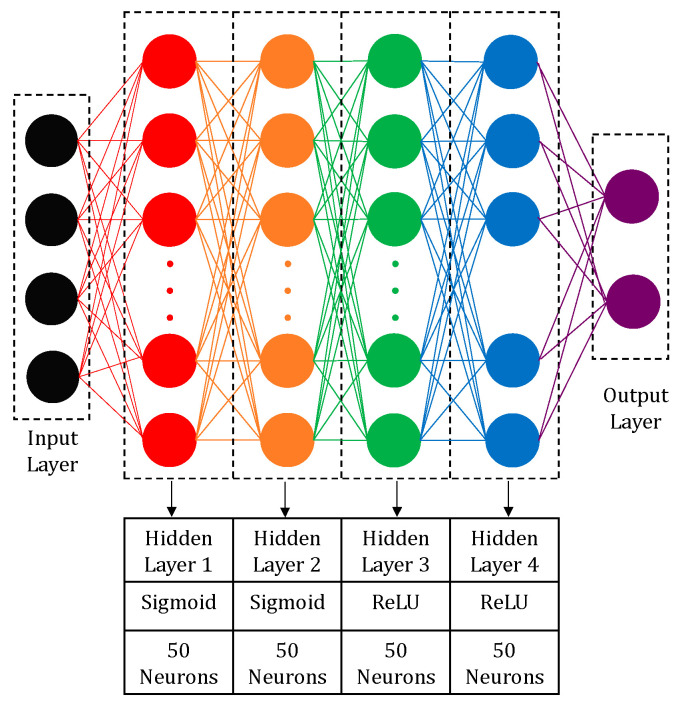
Deep neural network supervised training.

The deep learning neural network structure includes the depth, activation functions, and layer size. The shallow neural network (SNN) is presented in Figure 3 in order to compare with the developed deep learning models. Matlab R2019a (By PTC) software was implemented to program all the models. The structure of the deep neural network (DNN) is shown in Figure 4. The DNN structure has one input layer, four hidden layers, and an output layer. The laser power, scanning speed, hatching spacing, and island size are fed to the input layer. The output layer includes two nodes initiated by a sigmoid function, and the hardness and the porosity are the outputs. Each of the four hidden layers has 50 neurons. The Sigmoid function was employed to initiate the first and the second hidden layers, whereas the rectified linear unit function was employed to activate the third and the fourth layers, see Equations (2) and (3).

(2) $$\mathrm{Sigmoid}=\frac{1}{1+e^{-x}}$$

(3) Rectified Linear Unit (ReLU)=max(0,x)

where x is the function input.

Before training, the inputs and targets are normalized so that they always fall within a specified range. In this paper, the Matlab ‘’mapminmax’’ function was used to scale the inputs and the targets so that they fall in the range [0, 1]. Each node in the input layer is designated to a certain input. After training, the neural network model inputs and outputs are converted back into the same units that were utilised originally using Matlab “reversemapminmax” function.

**Figure 5:**
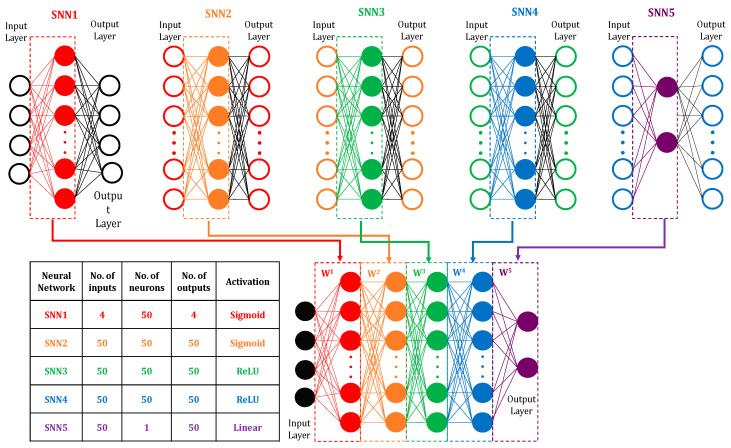
Deep learning neural network unsupervised greedy layer-wise pre-training approach.

An unsupervised greedy layer-wise pre-training is used to initialise the weights of the network, Figure 5. The deep learning model is pre-trained in five stages, where the non-input layers are trained sequentially using a shallow neural network. Although pre-training initialises the DLNN weights, it develops a sub-optimality [44]. Therefore, A fine-tuning is carried out to avoid any sub-optimality using a backpropagation technique [27]. In this algorithm, if the training pairs (*A1, t1*),…, (*An, tn*), where *As, 1 ≤ s ≤ n*, are the sth input, and *ts, 1 ≤ s ≤ n*, is the target, the least-square cost function is:

## 3. Results and Discussion

(4) $$L=\frac{1}{nZx}\overset{n}{\underset{s=1}{\sum }}{\left[t_{s-}O_{s}^{X}\right]}^{\tau }\left[t_{s-}O_{s}^{X}\right]$$

where $O_{s}^{X}$ is the output vector of the X-layered with *A_s_* as input, and *Z_X_* is the number of the output neurons.

Backpropagation [45] is a supervised learning technique (training algorithm) for neural networks, where the differences between the target outputs and neural predictions are employed to adjust the internal weights. Deep learning neural networks (DLNNs) is the structure of the networks. Networks with several hidden layers are referred to as DLNNs. Let *W* is a vector created by the network weights and ∇*E*(*W*(*k*)) is the *E* derivative at *W* = *W*(*k*), and *k* is the number of iterations of the weights vector. The backpropagation approach with a momentum term can be given as:

(5) ΔW(k)=α(−∇E(W(k)))+βΔW(k−1)

where α is the learning rate, β is the momentum factor, and Δ*W*(*k*) =*W*(*k* + 1) − *W*(*k*).

### 3.1. Optimisation of Deep Learning (DL) Models

The experimental matrix and the measured properties of the LPBF Ti-6Al-2Sn-4Zr-6Mo samples are shown in Table 2. The four process parameters are laser power, laser speed, hatching spacing, and island size, whereas the corresponding measured outputs are the porosity level and Vickers microhardness. As shown in the table, the porosity level and Vickers microhardness of the manufactured Ti-6Al-2Sn-4Zr-6Mo samples were found in a range of 0.07–1.04% and 330–441, respectively.

An automatic search for the optimal deep learning model was carried out by varying the initial number of layers, random seeds, number of neurons, and the activation functions. Several DNN structures with three, four, and five layers were trained and assessed. The structure presented in Figure 4 shows the lowest mean absolute error. Comparisons between unsupervised greedy layer-wise pre-training and backpropagation of the developed structure were carried out. The best model was then compared against DNN and SNN. The DNN model was chosen to be the same as the optimum DLNN. Table 3 shows the mean percentage error (MPE) for the tested approaches. The structure of the deep learning neural network (DLNN), in Figure 3, gave the lowest error compared to other models.

### 3.2. Validation

**Figure 6:**
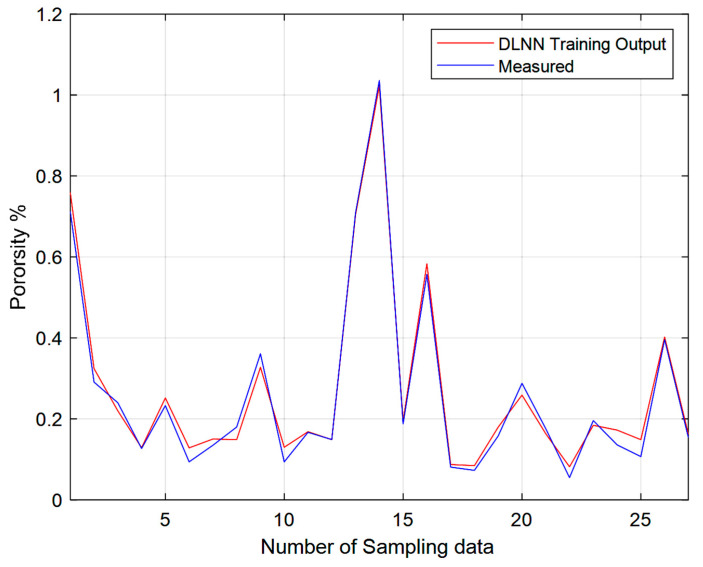
Measured porosity compared to the output of the DLNN model.

**Figure 7:**
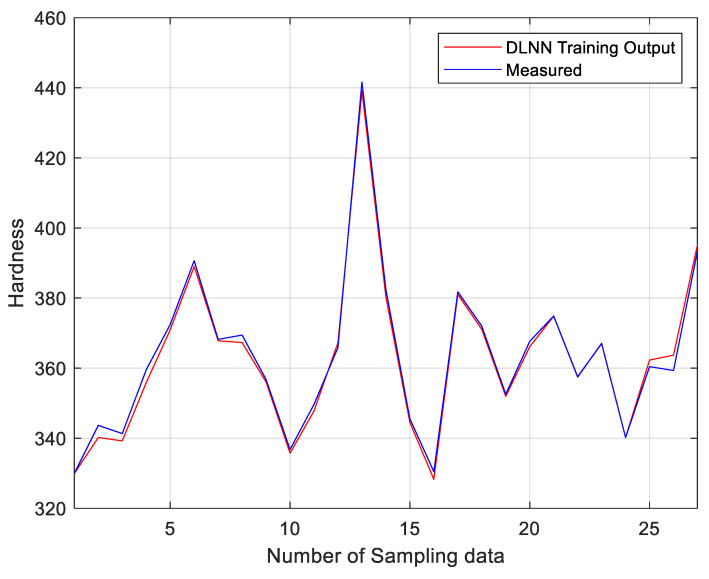
Measured hardness compared to the output of the DLNN model.

The mean percentage error results shown in Table 3 represents the model predictions using only 90% of the experimental data. For validation, a comparison between the model developed using deep learning neural network unsupervised greedy layer-wise pre-training approach and the 100% of the measured data of both the porosity and the hardness are shown in Figure 6 and Figure 7 respectively. The two figures show a strong agreement between the measured data and the DLNN model, represented by the red and the blue lines.

### 3.3. Microstructural Analysis

**Figure 8:**
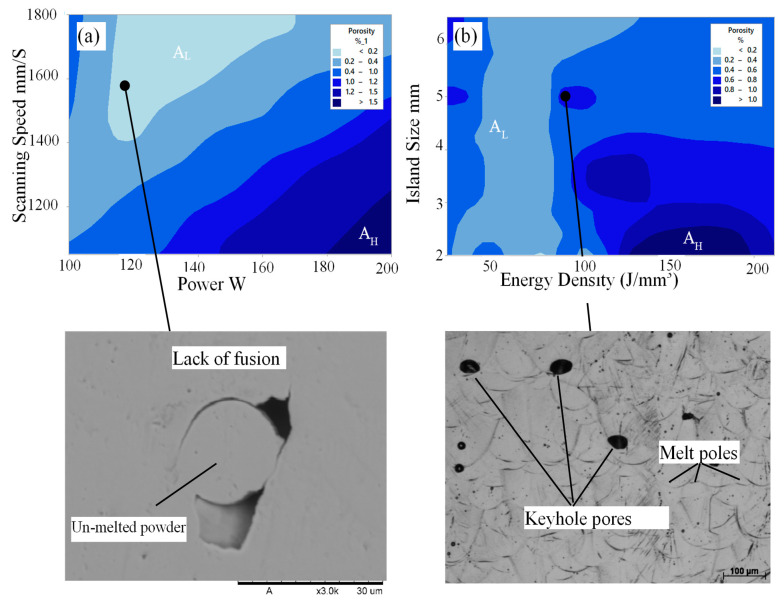
The predicted contours distribution of the porosity of Ti-6Al-2Sn-4Zr-6Mo alloy with respect to the process parameters and their corresponding porosity defects (**a**) effect of power and scanning speed, (**b**) effect of energy density and island size).

The optimised DLNN model was employed to develop a contour map of the porosity alloy with respect to the process parameters, see Figure 8. The contour distribution shows areas with the lowest and highest porosity, and they are indicated by AL and AH. Defects such as lack of fusion porosity and keyholes were detected in the microstructure of the samples. At low energy density, melt pools do not overlap, leaving unmelted powder and forms lack of fusion defects, while at high-energy input, melt pools become unstable and deepen, creating keyholes pores. It was also noted that the island size has an effect on the porosity of the sample when the energy density is high, though its original purpose was to distribute the heat energy across the part cross-section evenly and hence minimise the developed thermal stresses.

**Figure 9:**
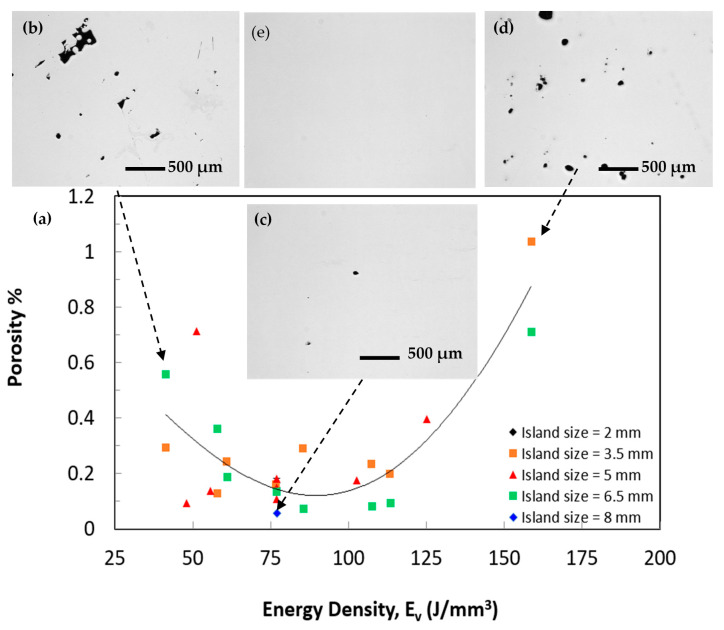
(**a**) Porosity level as a function of laser energy density (**b**–**e**) OM images of the samples fabricated using (**b**) Ev of 41 J/mm^3^, (**c**) Ev of 77 J/mm^3^, (**d**) Ev of 158 J/mm^3^, (**e**) HIPed post processing.

**Figure 10:**
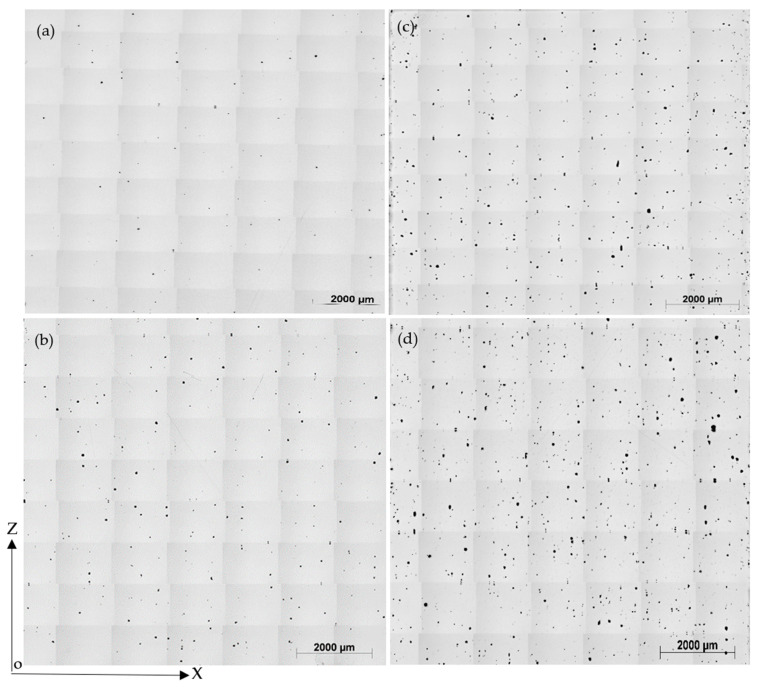
The variation of porosity level with island size for the same energy density, (**a**) Ev of 85 J/mm^3^, IS = 6.5 mm, Af = 0.07%; (**b**) Ev of 85 J/mm^3^, IS = 3.5 mm, Af = 0.29%; (**c**) Ev of 158 J/mm^3^, IS = 6.5 mm, Af = 0.71%; (**d**) Ev of 158 J/mm^3^, IS = 3.5 mm, Af = 1.04.

For the relation between the measured porosity of the Ti-6Al-2Sn-4Zr-6Mo bars and the volumetric energy density, see Figure 9. One of the shortcomings of the laser energy density model is its inability to explain the variation in densification behaviour for the samples processed using the same energy density, Figure 10. As shown, only one sample has a porosity percentage higher than 1%, which means that this alloy reacts very well to the laser. It can also be clearly seen that the laser energy density shows a strong influence on the porosity of the sample. This result is in agreement with reported research papers on the processing of titanium alloys using LPBF. Read et al. studied the effect of LPBF parameters on the porosity of AlSi10Mg alloy. The authors found that low energy density produces high porosity because of the reduced consolidation of the metal powder [46]. The porosity is then reduced by increasing the volumetric energy density. As the energy density increases further, the porosity increases again, similar to the contour map found in Figure 8b. In this region, defects such as keyhole pores were created within the microstructure of AlSi10Mg alloy, also similar to the SEM image shown in Figure 8b. El-Sayed et al. studied the volumetric energy density model’s effect on different properties of the porosity of Ti64 alloy, such as porosity content and modulus of elasticity [47].

Again, both low and high volumetric energy densities increase the porosity, whereas an optimum intermediate energy density significantly reduces the porosity content. Although a scatter was observed in Figure 9, the trained DLNN model showed a good agreement with the measured results with only 3% MPE, Figure 6 and Table 3. Overall, Ti-6Al-2Sn-4Zr-6Mo samples fabricated using E_v_ of 77–113 J/mm^3^ had relatively small porosity content, Figure 9c. Samples manufactured using E_v_ of 77 J/mm^3^ achieved the lowest porosity content of 0.07% and the lowest number of pores of 117. The porosity content increased as the E_v_ decreased from 77 J/mm^3^ or further increased from 113 J/mm^3^, Figure 9a. On the other hand, samples fabricated using E_v_ 51 J/mm^3^ shows a porosity level of 0.71% and a number of pores of 2043. Irregularly shaped porosity was found across the sample, which resulted from incomplete melting of the powders because of insufficient energy during the laser scanning of that area, Figure 9b.

Furthermore, samples fabricated using E_v_ 158 J/mm^3^ shows the highest porosity level of 1.036% and the largest average pore diameter of 615 µm. Generally, spherical pores can be attributed to entrapped gas within the gas atomised powder particles and keyhole defects, while irregularly shaped porosity resulted from incomplete melting of the powders because of insufficient energy during the laser scanning of that area, Figure 9d. In agreement with the DLNN prediction shown in Figure 8, samples produced using an island size of 6.5 mm achieved a lower porosity than those with 3.5 mm, at the same energy density, see Figure 10. This may be because many pores are formed at the interface between islands, which means that smaller islands would increase porosity due to the increase of island interfaces. In contrast to island size, we could not find a correlation between the samples’ porosity levels fabricated using the same energy density while varying the laser power, speed, or hatching space.

**Figure 11:**
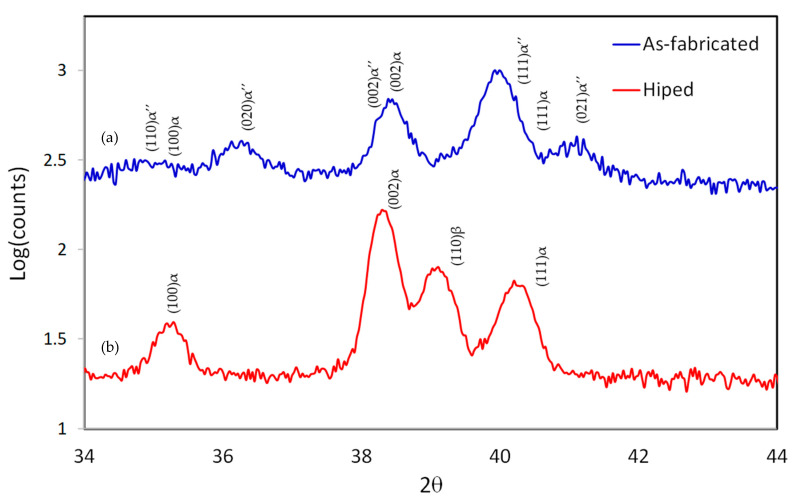
XRD results for samples fabricated using: (**a**) LPBF only; (**b**) LPBF followed by HIP process.

**Figure 12:**
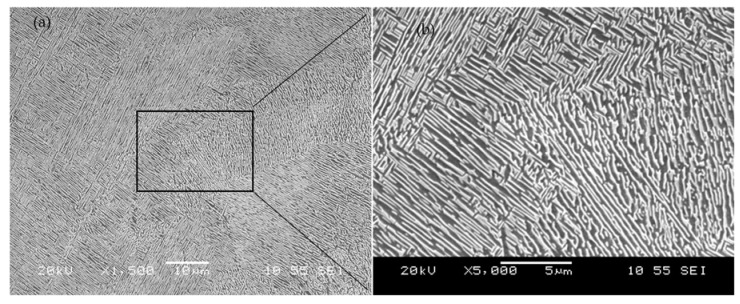
(**a**) Low and (**b**) high magnification SEM micrographs of the HIPed samples.

The XRD results of the LPBF and the HIPed samples are shown in Figure 11. α’’martenstic phase strong peaks while much less pronounced peaks of α can be found in the LPBF samples. The typical microstructure for a Ti-6Al-2Sn-4Zr-6Mo alloy produced using conventional techniques consists of α and β phases. The formation of the α’’martenstic phase is due to the laser energy input, which creates temperature above the β transus followed by rapid cooling. α’’martenstic phase is not an acceptable phase for industrial applications as it is brittle. Therefore, it is important to achieve a homogenous and stable microstructure as it affects the mechanical properties of a material. Controlling the microstructure can be achieved through post-processing treatments such as annealing or hot isostatic pressing (HIP). Figure 12a,b show the low and high magnification SEM micrographs of sample after the HIP post-processing. The microstructure and the XRD revealed the presence of α and β phases. The light regions are α phases, while the dark regions are the β phase. Using image analysis similar to the porosity calculation, the α phase fraction after HIP was 26.5% while the β phase was 73.5%.

### 3.4. Hardness

**Figure 13:**
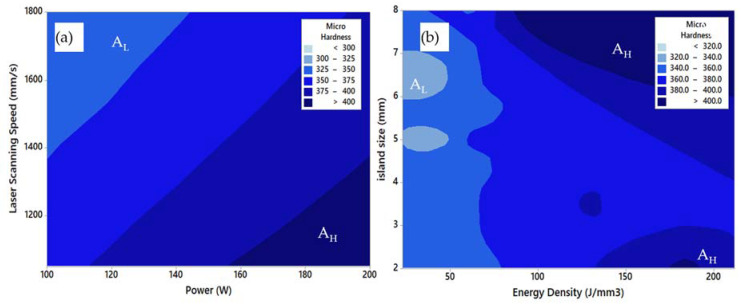
The predicted contours distribution of Vickers microhardness of Ti-6Al-2Sn-4Zr-6Mo alloy, (**a**) effect of power and laser scanning speed, (**b**) effect of energy density and island size.

Figure 13 shows the predicted contour map of the hardness against the process parameters using the optimum DLNN model. Similar to porosity, the effect of the island size parameter was found significant to the hardness.

**Figure 14:**
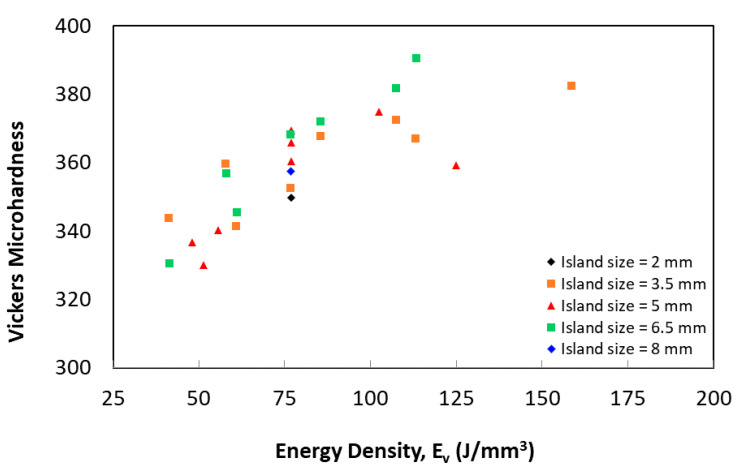
Vickers microhardness as a function of laser energy density.

Figure 14 shows the measured hardness with respect to energy density. A notable scatter is observed in most of the measurements. Although a hardness dataset was scattered along with the energy data, the model predicted it accurately, see Figure 7 and Figure 14. The highest Vickers microhardness was found in sample 13, which was fabricated using Ev 158 J/mm^3^ and an island size of 6.5 mm, while the lowest Vickers microhardness was obtained in sample 1, which was built using Ev 51 J/mm^3^ and island size of 5 mm. It was also found that island size has a contribution to the hardness measurement fluctuation of the samples fabricated using similar energy input, which may be attributed to the variation of the porosity content. This agrees with the predicted DLNN model, as shown in Figure 14. Samples fabricated using an island size of 6.5 mm achieved a higher hardness than those with 3.5 mm, at Ev > 61 J/mm^3^.

## 4. Conclusions

Laser powder fusion processing of Ti-6Al-2Sn-4Zr-6Mo as a popular material for aerospace and biomedical applications was presented in this paper to cover the literature gap in processing this alloy compared to other titanium alloys. The effect of LPBF parameters such as laser power, scanning speed, island size, and hatching spacing on the alloy porosity, hardness, and microstructure developments were investigated. Within the used processing parameters window, the Ti-6Al-2Sn-4Zr-6Mo alloy shows good processability using the LPBF system. The porosity level of the as-fabricated Ti-6Al-2Sn-4Zr-6Mo is generally low within the chosen process parameters ≈ 1%. Minimum porosity (<0.1) and a minimum number of pores can be achieved using a volumetric energy density of 77–113 J/mm^3^. The porosity content increased as the volumetric energy density decreased from 77 J/mm^3^ or further increased from 113 J/mm^3^. The Hardness values of the LPBF samples increased as volumetric energy density increased. The highest hardness was achieved using a volumetric energy density of 158 J/mm^3^ and an island size of 6.5 mm, while the lowest hardness was obtained when using volumetric energy density 51 J/mm^3^ and an island size of 5 mm. In addition, the results show that there was a notable fluctuation in the porosity and hardness measurements as the island size changes for samples built with the same volumetric energy density levels. The microstructural analysis reveals the presence of the undesirable α’’martensitic phase, which may be due to the laser energy input and the rapid cooling. The hot isostatic pressing was able to eliminate the porosity, α’’martensitic phase, and recover the desirable α and β phases. The relation between processing parameters and porosity and hardness measured data were determined using deep learning models. The DLNN model was the most accurate model and exhibited the lowest mean error percentage when compared to SNN and DNN. The developed DLNN model has the ability to predict the developed porosity content with an accuracy of 97% and a hardness of 99.8%.

## Figures and Tables

**Table 1:** The designed process parameters.

Process Parameter	Units	Levels				
		−2	−1	0	1	2
**Laser power**	W	100	125	150	175	200
**Scan speed**	mm/s	800	1050	1300	1550	1800
**Hatch spacing**	(*h*_1_)	0.2	0.35	0.5	0.65	0.8
**Island size**	mm	2.0	3.5	5.0	6.5	8.0

**Table 2:** The input parameters and response results.

Samples	Laser Power (W)	Scan Speed (mm/s)	Hatch Spacing (*a*_1_)	Island Size (mm)	Porosity %	Vickers Micro Hardness
1	100	1300	0.5	5	0.65 ± 0.03	330.0 ± 5.5
2	125	1550	0.65	3.5	0.3 ± 0.02	343.7 ± 5.1
3	125	1050	0.65	3.5	0.25 ± 0.02	341.3 ± 5.4
4	175	1550	0.65	3.5	0.13 ± 0.02	359.6 ± 8.6
5	175	1550	0.35	3.5	0.25 ± 0.02	372.4 ± 6.5
6	125	1050	0.35	6.5	0.19 ± 0.02	390.7 ± 9.8
7	125	1550	0.35	6.5	0.13 ± 0.01	368.2 ± 5.7
8	150	1300	0.5	5	0.18 ± 0.02	369.4 ± 5.6
9	175	1550	0.65	6.5	0.37 ± 0.02	356.8 ± 4.6
10	150	1300	0.8	5	0.11 ± 0.01	336.8 ± 5.3
11	150	1300	0.5	2	0.17 ± 0.01	349.7 ± 8.2
12	150	1300	0.5	5	0.16 ± 0.01	365.9 ± 4.0
13	175	1050	0.35	6.5	0.74 ± 0.03	441.7 ± 6.8
14	175	1050	0.35	3.5	1.04 ± 0.04	382.4 ± 10.2
15	125	1050	0.65	6.5	0.19 ± 0.01	345.6 ± 6.5
16	125	1550	0.65	6.5	0.54 ± 0.03	330.4 ± 6.3
17	175	1550	0.35	6.5	0.09 ± 0.01	381.8 ± 3.1
18	175	1050	0.65	6.5	0.08 ± 0.01	372.0 ± 9.5
19	125	1550	0.35	3.5	0.16 ± 0.01	352.6 ± 6.9
20	175	1050	0.65	3.5	0.28 ± 0.02	367.6 ± 9.8
21	200	1300	0.5	5	0.19 ± 0.01	374.9 ± 6.0
22	150	1300	0.5	8	0.07 ± 0.01	357.6 ± 5.9
23	125	1050	0.35	3.5	0.18 ± 0.01	367.0 ± 6.7
24	150	1800	0.5	5	0.16 ± 0.01	340.2 ± 6.5
25	150	1300	0.5	5	0.15 ± 0.01	360.4 ± 8.6
26	150	800	0.5	5	0.4 ± 0.02	359.3 ± 8.2

**Table 3:** Mean percentage error comparison for tested approaches.

Approach	Porosity	Hardness
DLNN	3%	0.3%
DNN	18%	12%
SNN	46%	36%

## Data Availability

Data is contained within the article.

## Nomenclature

AM	Additive manufacturing
α	learning rate
*A_s_*	Input neurons
β	momentum factor
b	powder layer thickness
DL	deep learning
DOE	design of experiments
DLNNs	deep learning neural networks
*E*	volumetric energy density
h	hatching spacing
k	number of iteration of the weights vector
LPBF	Laser powder bed fusion
OM	optical microscope
$O_{s}^{X}$	output vector of the X-layered
P	laser power
L	The least-square cost function
ReLU	Rectified Linear Unit
SNN	Shallow neural network
s	laser speed
SEM	electron scanning microscopy
W	vector
x	the function input
*Z_X_*	output neurons number
